# WNT ligands control initiation and progression of human papillomavirus-driven squamous cell carcinoma

**DOI:** 10.1038/s41388-018-0244-x

**Published:** 2018-04-17

**Authors:** Dario Zimmerli, Virginia Cecconi, Tomas Valenta, George Hausmann, Claudio Cantù, Gaetana Restivo, Jürg Hafner, Konrad Basler, Maries van den Broek

**Affiliations:** 1 Institute of Molecular Life Sciences, University of Zürich, 8057 Zurich, Switzerland; 2 Institute of Experimental Immunology, University of Zürich, 8057 Zurich, Switzerland; 3 Department of Dermatology, University Hospital Zürich, 8091 Zürich, Switzerland; 4 Present Address: Department of Clinical and Experimental Medicine (IKE), Faculty of Health Sciences, Wallenberg Center for Molecular Medicine (WCMM); Linköping University, S-58185 Linköping, Sweden

## Abstract

Human papillomavirus (HPV)-driven cutaneous squamous cell carcinoma (cSCC) is the most common cancer in immunosuppressed patients. Despite indications suggesting that HPV promotes genomic instability during cSCC development, the molecular pathways underpinning HPV-driven cSCC development remain unknown. We compared the transcriptome of HPV-driven mouse cSCC with normal skin and observed higher amounts of transcripts for Porcupine and WNT ligands in cSCC, suggesting a role for WNT signaling in cSCC progression. We confirmed increased Porcupine expression in human cSCC samples. Blocking the secretion of WNT ligands by the Porcupine inhibitor LGK974 significantly diminished initiation and progression of HPV-driven cSCC. Administration of LGK974 to mice with established cSCC resulted in differentiation of cancer cells and significant reduction of the cancer stem cell compartment. Thus, WNT/β-catenin signaling is essential for HPV-driven cSCC initiation and progression as well as for maintaining the cancer stem cell niche. Interference with WNT secretion may thus represent a promising approach for therapeutic intervention.

## Introduction

Cutaneous squamous cell carcinoma (cSCC) develops from basal keratinocytes in sunlight-exposed skin and is the second most frequent cancer in fair-skinned individuals. HPV-driven cSCC is the most common cancer in immunosuppressed organ transplant recipients (OTR), up to 50% of those patients develop cSCCs within 10 years after transplantation [1]. Thus, immunosuppression increases the risk to develop cSCC by 250-fold [2, 3]. In addition, 80% of cSCC in OTR are positive for HPV compared with 40% in non-OTR [4], suggesting a role for HPV in the initiation and/or progression of cSCC particularly in immunosuppressed individuals. This is underscored by a recent report showing that vaccination against HPV protects against keratinocyte-derived cancers [5]. Although the main driver mutations of HPV-negative cSCCs are found in the RAS-MAPK signaling pathway [6], a role for WNT/β-catenin signaling has been reported as well [7].

The WNT/β-catenin signaling cascade is essential in many developmental processes but is also involved in initiation and progression of many cancer types. Signaling is initiated by the binding of secreted WNT ligands (WNTs) to the Frizzled/LRP receptor complexes. Secretion of WNT ligands fully depends on their acylation by the acyltransferase Porcupine (PORCN) [8, 9]. Binding of WNTs to Frizzled/LRP triggers a cascade of events, which culminates in cytoplasmic stabilization and subsequent nuclear translocation of β-catenin. In the nucleus, β-catenin associates with TCF/LEF transcription factors to drive expression of WNT target genes [10]. WNTs also initiate various β-catenin independent outputs, some of which may play a role in cSCC [11].

To investigate the molecular mechanisms underlying the initiation and progression of HPV-driven cSCC, we used a mouse model in which UV-induced overexpression of the HPV8-derived E6 oncogene in keratinocytes drives the development of a progressive cSCC (*Krt14*-HPV8(*E6*); [12, 13]). We found increased expression of WNTs and PORCN in cSCCs. Furthermore, blocking WNT secretion by a small-molecule inhibitor of PORCN, LGK974 decreased the cSCC stem cell compartment and inhibited induction and progression of cSCC.

## Results and discussion

### Increased expression of WNT ligands and elevated WNT/β-catenin signaling are hallmarks of HPV8-E6 driven cSCC

**Fig. 1:**
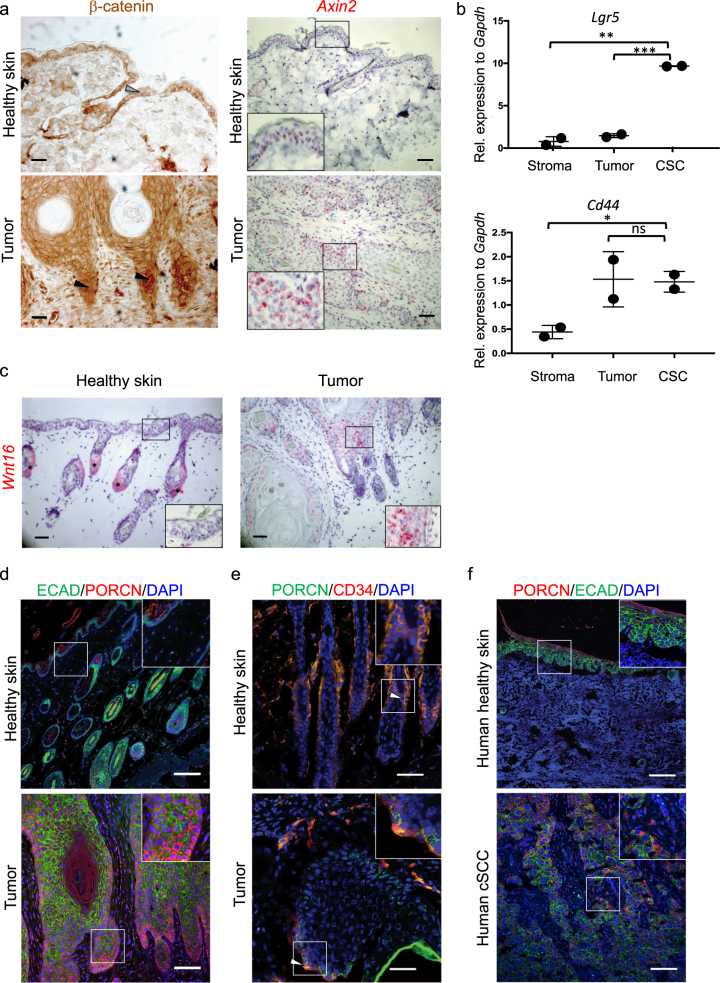
Elevated WNT/β-catenin signaling and increased expression of WNT ligands are hallmarks of HPV8-E6 driven cSCC. **a** Left panel: nuclear accumulation of β-catenin in the invasive front of HPV-driven cSCCs (black arrowheads). Healthy skin shows membranous expression of β-catenin (gray arrowhead). Scale bar = 50 µm. Right panel: *in situ* hybridization for *Axin2* (red stain) shows increased expression of this WNT/β-catenin signaling target in tumors in comparison with healthy skin. Nuclei are counterstained with hematoxylin (blue stain). Samples were collected 4 weeks after tumor induction. Scale bar = 100 μm. Representative samples of a total of four biological replicates are depicted. **b** Enhanced amount of *Lgr5* transcripts in CSCs (EpCAM^+^ CD34^+^ CD49f^+^) compared to non-CSC tumor cells (EpCAM^+^ CD34^-^) and stromal cells (EpCAM^−^ CD34^−^ CD49f^−^) (upper panel). The Wnt target gene *Cd44* shows low expression in the stroma and high expression in all epithelial tumor cells (lower panel). Cells from HPV-driven cSCC from two different mice were sorted and expression was quantified by qRT-PCR. Samples were collected 4 weeks after tumor induction. Symbols represent individual mice. This experiment was done once. **c** *In situ* hybridization of healthy skin and HPV-driven cSCC for *Wnt16* (red), shows higher expression of *Wnt16* in tumors compared with healthy skin. Asterisks marks unspecific chromogen accumulation in sebaceous gland cells. The section is counterstained with hematoxylin. Scale bar = 50 µm. Samples were collected 4 weeks after tumor induction. Representative samples of a total of three biological replicates are depicted. **d** PORCN (red) staining in healthy skin and HPV-driven cSCC. E-cadherin (green) marks epithelial cells, DAPI (blue) marks nuclei. Scale bar = 100 μm. PORCN is mostly expressed in tumor epithelial cells along the tumor–stroma interphase of the tumor, whereas it is absent from healthy epidermis. Insets show a larger magnification of the region marked by the white square. Samples were collected 4 weeks after tumor induction. Representative samples of a total of four biological replicates are depicted. **e** Overlapping expression of PORCN (green) and CD34 (red) in healthy skin with hair follicles and tumor tissue is indicated by arrowheads. Scale bar = 50 μm. Insets show a larger magnification of the region marked by the white square. Samples were collected 4 weeks after tumor induction. Representative samples of a total of three biological replicates are depicted. **f** Increased expression of PORCN (red) in human cSCC in comparison with healthy human skin. E-cadherin (green) marks epithelial tissue, nuclei are counterstained in blue. Scale bar = 100 μm. Insets show a larger magnification of the region marked by a white square. Additional pictures are shown in Supplementary Figure 3. A representative sample of a total of nine is depicted. *Krt14*-HPV8*(E6)* mice were described previously [13] and were bred to FVB mice (Harlan Laboratories, Envigo) in house. Mice were kept under specific pathogen-free conditions at the Laboratory Animal Services Center at the University of Zurich and 6–8-weeks-old, sex-matched mice were used for all experiments. To induce cSCC, ~ 4 cm^2^ of shaved dorsal skin was irradiated with UVA (5 J/cm^2^) plus UVB (1 J/cm^2^) using the UV 802 L Waldmann device. Murine tumor samples were collected 4 weeks after tumor induction. Experiments were performed in accordance with the Swiss federal and cantonal regulations on animal protection and were approved by the Cantonal Veterinary Office Zurich. The Swiss law on animal protection demands that groups sizes are as small as possible. The clear biological differences allowed statistical differences with small group sizes. No animals were excluded from analysis in any experiment. In case of treatment of mice with established tumors, mice were randomized in two groups before start of treatment based on tumor size; in all other cases mice were not randomized. The investigators were not blinded. Groups were compared with an unpaired Student’s *t*-test and show the mean ± SD. *p < 0.05, **p < 0.01, ***p < 0.001. Based on comparable SD similar variance was assumed. The above information applies to all animal experiments in this report. Human SCC samples (nine samples in total; three were dedifferentiated, five were moderately differentiated, one was well-differentiated) were obtained from biobanks managed by the University Research Priority Project “Translational Cancer Research” and the research project “Skintegrity”. All material were surplus biopsies from patients who had signed an informed consent that was approved by the Cantonal Ethics Commission Zurich (EK647 and EK800). For flow cytometry and sorting, tumors were collected in PBS and digested for 2 h at 37 °C in 2.4 mg/ml Dispase (Roche), cut into pieces and digested again for one hour at 37 °C in 1 mg/ml collagenase Type IV (Worthington Biochemical Corporation) and 0.1 mg/ml DNase (Sigma-Aldrich). Antibodies against the following proteins were used: CD45.1 (clone A20, BioLegend), CD31 (clone MEC13.3, BioLegend), EpCAM (clone GoH3, BioLegend), CD34 (clone RAM34, eBioscience). Dead cells were excluded using Zombie Violet Fixable Viability Kit (BioLegend). Doublets were excluded by FSC-A versus FSC-H and SSC-A versus SSC-H gating. Immunohistochemistry on frozen sections was performed on tissue fixed in 4% PFA for 1 h, left to sink in 30% sucrose and embedded in OCT. Ten-µm-thick cryosections were blocked for 1 h at RT with 2.5% Hings and 2.5% BSA in 0.1% Tween in PBS (PBST) (blocking buffer), and then stained with biotin-conjugated anti-CD34 (clone RAM34, eBioscience) and unconjugated rabbit anti-PORCN (clone ab105543, Abcam) overnight at 4 °C in blocking buffer. Secondary antibodies (see below) were added for 1 h in blocking buffer at RT, then samples were mounted in FluorSave (CalBiochem). Standard protocols were used for embedding and cutting formalin-fixed paraffin-embedded (FFPE) tissue. After deparaffinization, on both mouse and human samples, antigen retrieval was performed in 10 mM trisodium citrate buffer pH 6. Staining was performed as described above for frozen sections. The following antibodies were used for mouse and human FFPE samples: Mouse-anti-β-catenin (clone14, BD transduction labs), mouse-anti-E-cadherin (BD transduction labs), rabbit anti-PORCN (Abcam ab105543), rabbit anti-Ki67 (Abcam), rabbit anti-HMGA2 (SantaCruz), rabbit anti-MMP13 (clone 3H13L17, ThermoFisher), and rabbit anti-phospho-ERK (Cell Signaling). Secondary antibodies used were goat-anti-mouse AlexaFluor 488 and goat-anti-rabbit AlexaFluor 555. For the PORCN staining a biotin-labeled goat-anti-rabbit secondary antibody was used, followed by the ABC kit (VectaShield), and the Cy3 tyramide amplification kit (PerkinElmer). Staining for nuclear β-catenin was performed with biotin-labeled secondary antibodies, followed by DAB staining (VectorLabs). When mouse primary antibodies were used on murine tissues, VectorLabs MOM kit was used to block endogenous antigens. *In situ* hybridization was performed using the RNAscope kit (Advanced Cell Diagnostics) according to the manufacturer’s instructions. Probes for *Axin2* and *Wnt16* were obtained from the same company. For qRT-PCR, RNA was isolated from sorted cells using the NucleoSpin RNA XS kit (Machery-Nagel) according to the manufacturer’s instructions. qRT-PCRs using SybrGreen were performed on cDNA synthesized with the Roche Transcriptor High Fidelity cDNA Synthesis Kit after RNA isolation by standard TRI-Reagent protocols. Reactions were performed in triplicates and monitored with the ABI Prism 7900HT system (Applied Biosystems). The following 5’–3’ primers (Microsynth) were used for qRT-PCR. *Gapdh*, fwd AACTTTGGCATTGTGGAAGG, rev ATCCACAGTCTTCTGGGTGG; *Lgr5*, fwd CTCCACACTTCGGACTCAACAG, rev AACCAAGCTAAATGCACCGAAT

Aberrantly active WNT/β-catenin signaling drives various epithelial cancers, including non-viral cSCC [14-16]. The importance of WNT/β-catenin signaling in HPV-driven tumors is unknown. To investigate this, we used the *Krt14*-HPV8(*E6*) transgenic mouse model for cSCC in which tumor induction can be triggered by UV-irradiation [13]. We quantified stabilization of β-catenin and observed an increased ratio of intracellular (nuclear as well as cytoplasmic) β-catenin over membranous β-catenin in SCC compared to healthy skin (Fig. 1a, left panels, supplementary Figure 1a, b); increased intercellular presence of β-catenin is an accepted hallmark for active WNT/β-catenin signaling [10]. We assume that different densities of stromal cells in wild type skin and tumor samples are responsible for the increased background in stromal tumor tissue. We confirmed elevated WNT/β-catenin signaling by *in situ* hybridization for the WNT/β-catenin target gene *Axin2* [17] (Fig. 1a, right panels, supplementary Figure 1c).

In non-viral cSCCs, cancer stem cells (CSC) reside at the tumor–stroma interface in close proximity to the vasculature [18] and express, besides EpCAM, several integrins including integrin β1 (CD29) and α6 (CD49f) [19] as well as CD34 [14, 19, 20]. Indeed, we found a putative population of CSCs that were CD34^+^ at the tumor–stroma interface in HPV-driven cSCCs (Supplementary Figure 1d). SCC-associated CSC often lose epithelial markers and would therefore not necessarily express KRT6 [21]. In addition, vascular cells can express CD34 [22], explaining the positive cells far away from the tumor–stroma interphase. To strengthen the point that we indeed find CD34 positive CSCs as described for SCC [19], and that CD34 expression can be used to identify SCC-associated CSCs in the experimental model, we performed co-staining of CD34 and E-cadherin and found double positive cells in a subset of CD34-positive cells at the tumor–stroma interphase (Supplementary Figure 1d).

Importantly, flow cytometry analysis of tumors showed that EpCAM^+^ CD34^+^ CSCs expressed a higher amount of the WNT/β-catenin target gene and stem cell marker *Lgr5* [23], when compared with non-stem cell tumor cells (EpCAM^+^ CD34^-^) or stromal cells (EpCAM^−^CD34^−^) (Fig. 1b). Thus, elevated WNT signaling and expression of LGR5 seem to mark the CSC pool in analogy to what has been described for non-viral cSCC and colorectal cancer [14, 24].

To better understand the involvement of WNT/β-catenin signaling in HPV-driven cSCCs, we compared the transcriptome of established cSCC with that of healthy skin. Gene ontology analysis [25] suggested that differentially expressed genes are associated with increased cell motility, altered cell-matrix adhesion and inflammation (Supplementary Figure 2a). Several transcripts that have previously been associated with non-viral cSCC, such as *Fosl1* (encoding Fos-like antigen 1), *Ptprz1* (encoding protein tyrosine phosphatase, receptor type Z1) [19,21], as well as transcripts encoding for metalloproteases (*Mmp*), were also upregulated in HPV-driven cSCC (Supplementary Figure 2b), suggesting the existence of common pathways in cSCC that are independent of disease etiology.

Although no mutations in *Hras* were reported in the *Krt14*-HPV8(*E6*) cSCC tumor model [13], we found a significant upregulation of the RAS-MAPK-ERK signaling pathway (Supplementary Figures 2a and 2c). Therefore, RAS-MAPK-ERK activation seems to represent a common mechanism associated with cSCC progression together with elevated WNT/β-catenin signaling.

We did not detect an increase of expression of WNT/β-catenin signaling target genes. This was not unexpected because of the very high activity of the WNT pathway in hair follicles, especially during the hair growth (anagen) phase [26]. However, consistent with the presence of activated WNT/β-catenin signaling in HPV-driven cSCCs, we observed decreased expression of extracellular WNT-inhibitory factors, including members of the *Sfrp* (encoding secreted frizzled-like protein)-family, *Notum* and *Dkk* (encoding Dickkopf). We also observed increased expression of transcripts of some *Wnt*s and *Porcn*, whose product is essential for WNT secretion (Supplementary Figure 2d). Interestingly, there is also evidence that precise regulation of this enzyme is required for physiological WNT signaling [27]. Using *in situ* hybridization, we confirmed strong *Wnt16* expression in most epithelial tumor cells, whereas *Wnt16* mRNA was below detection levels in healthy skin. This confirms our RNA-sequencing results, where *Wnt16* was one of the most upregulated genes, and also shows that *Wnt16* is specifically upregulated in malignant but not normal skin epithelial cells (Fig. 1c). This is in agreement with the observation that elevated WNT16 expression correlated with enhanced WNT/β-catenin signaling and consequent cell survival and therapy resistance in prostate cancer [28, 29].

Recent studies have highlighted the importance of PORCN for establishment and maintenance of a CSC niche [30]. We found that epithelial tumor cells at the tumor–stroma interface, which is where the CSCs reside, strongly expressed PORCN, whereas stromal cells rarely did (Fig. 1d). To confirm this, we stained for CD34 in concert with PORCN and found that the PORCN^+^ cells included CD34^+^ and CD34^-^ cells (Fig. 1e). Thus, PORCN expression may mark the WNT-rich CSC niche in cSCC as was described recently for lung adenocarcinomas [30].

To validate the clinical relevance of our findings, we investigated PORCN expression in human cSCC and observed increased expression in 6/6 samples independently of tumor grade (Fig. 1f, supplementary Figure 3a-f). Furthermore, we stained a commercially obtained tissue micro array (TMA) containing 76 biopsies of human SCC (20 stage 1; 53 stage 2; 3 stage 3) and four biopsies of healthy human skin for PORCN and E-Cadherin and assessed the presence of PORCN-positive epithelial cells. We detected PORCN expression in the majority of tumors, independently of the tumor stage. Specifically, 80% (17/20) of stage 1, 76% (40/53) of stage 2 and 100% (3/3) of stage 3 tumors expressed PORCN, whereas healthy skin was negative (supplementary Figure 3g). As ~20% of tumors in this TMA did not express PORCN, it might be interesting to compare the genetic landscape of such tumors to PORCN-positive tumors to understand whether PORCN-negative tumors are independent of WNT signaling. Of note, PORCN expression was mostly restricted to the invasive front of the tumors, suggesting a role for PORCN in CSC maintenance and tissue invasion in human cSCC too (Supplementary Figure 3).

### Inhibition of WNT secretion impairs the initiation of HPV-driven cSCC

**Fig. 2:**
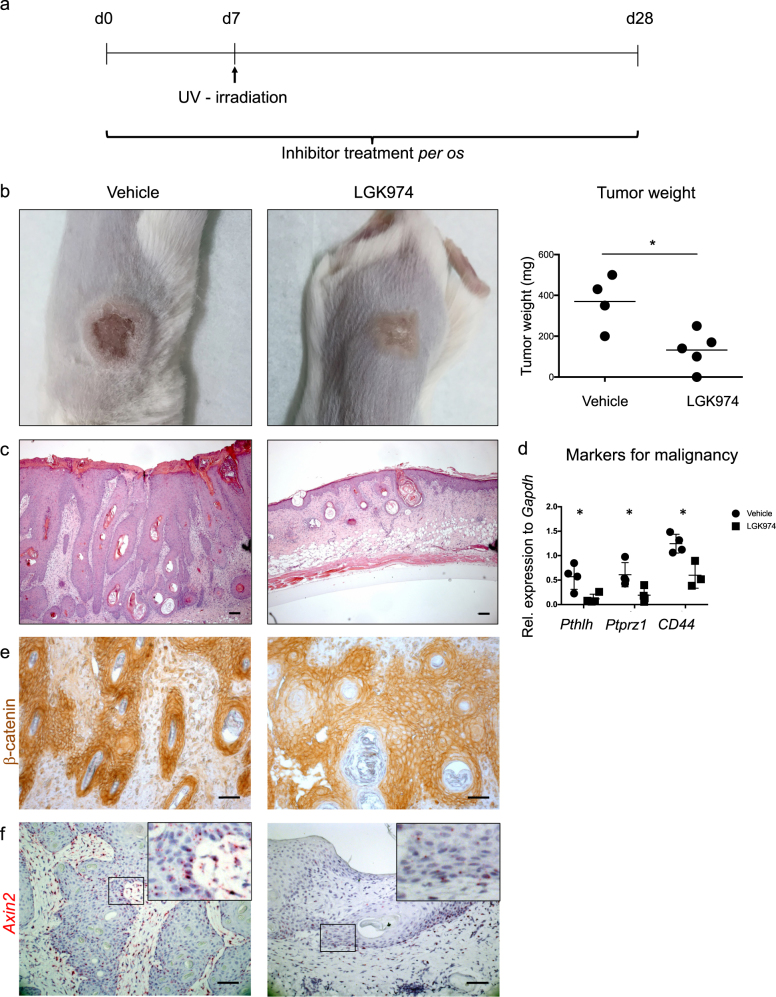
Inhibition of WNT secretion by the PORCN inhibitor LGK974 impairs the initiation of HPV-driven cSCC. **a** Experimental design. Treatment with LGK974 (6 mg/kg) or vehicle was started 7 days prior to tumor induction by UV-irradiation. Mice were treated daily until the end point at day 28. Control mice were treated with vehicle. The vehicle-treated group consisted of four, the LGK974-treated group of five mice. The experiment was performed twice with similar results. **b** Representative macroscopic display of a vehicle-treated (left panel) and LGK974-treated (middle panel) tumor. Tumor weight at endpoint (right panel). Symbols represent individual mice. **c** Representative H&E staining of vehicle-treated (left panel) and LGK974-treated (right panel) tumors. Scale bar = 100 µm. **d** Quantification of transcripts for *Pthlh*, *Ptprz1*, and *Cd44* in LGK974- and vehicle-treated tumors shows significant reduction of markers for tumor malignancy upon treatment. Symbols represent individual mice. **e** Representative staining showing stabilization of β-catenin in cytoplasm and nucleus of vehicle-treated tumors, whereas it is mostly membrane associated upon LGK974 treatment (Scale bar = 50 μm). **f** Representative image of *Axin2 in situ* hybridization showing reduced transcripts (red) upon LGK974- treatment. Nuclei are counterstained with Hematoxylin. Scale bar = 50 μm. The Porcupine inhibitor LGK974 was applied as described in ref. [31]. In brief, LGK974 was dissolved in DMSO and diluted in citrate buffer pH 3 to a final concentration of 1 mg/ml. Mice were treated daily with 6 mg/kg LGK974 or vehicle (20% DMSO in citrate buffer pH 3) *per os*. Staining and qPCR Protocols are described in the legend of Fig. 1. The primers used were *Pthlh*, fwd ATCCCCGACGCCTATGTAA, rev GGGGAAAAAGCAATCAGAGA; *Ptprz1*, fwd GCCAGTTGTTGTCCACTGC, rev CCTTTGAGAACGAATGTGCTT; *Cd44*, fwd CTCCTTCTTTATCCGGAGCAC, rev TGGCTTTTTGAGTGCACAGT

To address whether WNT secretion and -signaling are essential for the initiation of HPV-driven cSCC, we administered a small molecule inhibitor of PORCN (LGK974), which blocks the secretion of all WNTs [31]. LGK974 (or vehicle) was administered daily *per os*, starting 1 week prior to tumor induction (Fig. 2a). Administration of 6 mg/kg LGK974 or vehicle did not affect the general wellbeing of mice or the intestinal stem cell compartment, which is most sensitive to perturbations of WNT/β-catenin signaling [32], (data not shown).

Treatment with LGK974 prior to the tumor induction resulted in a significant reduction in tumor size in comparison to the vehicle-treated control mice (Fig. 2b), suggesting an essential role of PORCN and consequently of WNTs in the initiation of HPV-driven cSCC. Analysis of hematoxylin and eosin (H&E)-stained sections confirmed that UV-induced skin inflammation failed to progress to carcinoma in LGK974-treated mice, whereas characteristic cSCC lesions were observed in vehicle-treated mice (Fig. 2c). In contrast, the lesions in LGK974-treated mice (Fig. 2b) consisted mainly of keratin whorls reminiscent of differentiation and in some cases of hair follicles with normal morphology (Supplementary Figure 4a, b).

Expression of CSC markers is indicative for malignancy and loss of stem cells often results in differentiation and ultimately regression of tumors [33]. WNT/β-catenin signaling is one of the key pathways defining the stem cell niche in many epithelial tissues and cancers of epithelial origin [24, 34]. As a next step, we therefore investigated whether inhibition of WNT secretion has an impact on CSCs in HPV-driven cSCCs. Thus, we quantified transcripts by qRT-PCR that are expressed in cSCC stem cells [14, 19, 20]. We focused on *Pthlh* (encoding parathyroid hormone-like hormone), *Ptprz1* and *Cd44* because of their association with CSC in a chemically induced model of cSCC [19, 35, 36]. In addition, *Cd44* is a prominent Wnt target gene [37]. Blocking WNT secretion via LGK974 administration resulted in significantly reduced expression of all abovementioned CSC markers (Fig. 2d). Upon treatment with LGK974 we observed a reduction in intracellular β-catenin staining (Fig. 2e, supplementary Figure 4c), as well as a reduction of *Axin2* expression by *in situ* hybridization in regions lacking hair follicles (Fig. 2f, supplementary Figure 4d). We detected no changes in transcripts of the WNT target genes *Axin2* as well as *Lgr5* when using qRT-PCR, which may be explained by the increase in the number of hair follicles in anagen (Supplementary Figure 4a,b).

To confirm the specificity of PORCN inhibition, we used Wnt-C59 [38], another PORCN inhibitor, in the same pre-treatment experimental setup as described above and obtained similar results with respect to reduced tumor growth and inhibition of the Wnt/β-catenin pathway activation (Supplementary Figure 5a-d).

Together our data suggest that WNT ligands are essential for the stem cell niche in HPV-driven cSCCs. In support of these findings, inhibition of PORCN inhibited also basal cell carcinoma (BCC) [39] as well as keratoacanthoma development [40].

### WNTs promote progression of HPV-driven cSCC

As preemptive inhibition of WNT secretion significantly reduced the development of HPV-driven cSCC, we investigated whether blocking WNT secretion also interferes with tumor progression when started at the time of tumor induction or after tumors had established.

When we started treatment with LGK974 simultaneously with tumor induction, we observed increased differentiation and reduced thickness of the tumor upon treatment, but did not see complete inhibition of tumor growth (Supplementary Figure 6). A possible explanation for the inability to copy the results of the treatment starting before tumor induction with this experiment is the feature of WNT proteins to remain intact for an extended period of time [32].

**Fig. 3:**
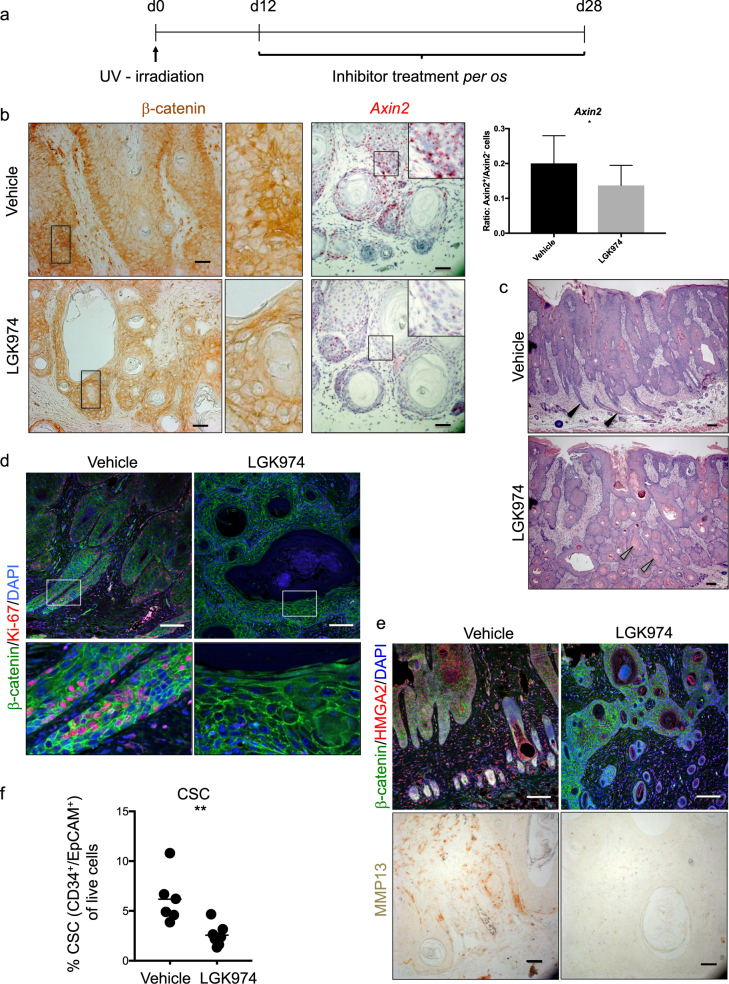
WNTs promote HPV-driven cSCC progression. **a** Experimental design. Treatment with LGK974 (6 mg/kg) was started 12 days after tumor induction. Mice were treated daily until the endpoint at day 28. Control mice were treated with vehicle. The vehicle-treated group consisted of five, the LGK974-treated group of six mice. The experiment was performed twice with similar results. **b** β-catenin staining of LGK974- and vehicle-treated tumors. The amount of β-catenin protein was reduced upon treatment. Middle panels show a magnification of the boxed area. Right panels show an *in situ* hybridization for *Axin2* (red), indicating a reduction of its expression upon treatment, nuclei are counterstained with hematoxylin (blue). Quantification was performed by the Vectra 3.0 system (PerkinElmer) and is shown in the right panel. Unpaired Student’s *t*-test, *p < 0.05 Scale bar = 50 µm. **c** Keratin whorls (gray arrowheads in lower panel) and loss of invasive cones (black arrowheads in upper panel) indicate differentiation in H&E-stained sections of vehicle- and LGK974-treated cSCC. Scale bar = 100 μm. **d** Sections stained for Ki67 (red) as marker for proliferation and β-catenin (green) to outline the cells, nuclei are counterstained by DAPI. The staining shows a clear reduction of proliferating Ki67-positive cells upon treatment. Lower panels show a magnification of the boxed area. Scale bar = 100 μm. **e** HMGA2 (red) is strongly reduced upon treatment in the tumors. β-catenin in green marks the cells, DAPI counterstains the nuclei in blue. Scale bar = 100 µm. MMP13 expression is lost upon LGK974 treatment. Scale bar = 50 μm. **f** Flow cytometric analysis of the percentage of CSC in comparison to live cells (EpCAM^+^ CD34^+^ CD31^−^) in vehicle- and LGK974-treated cSCC. Symbols represent individual mice. Protocols are described in the legend of Fig. 1. HMGA2 staining was performed as described for PORCN, MMP13 staining as described for nuclear β-catenin staining

Next, we treated established cSCC with LGK974 (Fig. 3a). We observed and quantified a marked reduction of β-catenin stabilization in response to LGK974 treatment, indicative of reduced WNT/β-catenin signaling. This finding was corroborated by a reduction in *Axin2* expression, especially at the tumor–stroma interphase (supplementary Figure 7a, Fig. 3b). Although LGK974-treament did not macroscopically reduce tumor size, analysis of H&E-stained sections revealed that LGK974-treated tumors displayed a more differentiated morphology than vehicle-treated tumors (Fig. 3c). A good indicator of reduced malignancy and the differentiation status of cSCCs is the increased presence of keratin whorls [41] and a reduced density of invasive cones [42]. LGK974-treated tumors showed both: an increased density of keratin whorls and a loss of invasive cones (Fig. 3c). Furthermore, and in line with increased differentiation, LGK974-treated tumor cells proliferated less than control tumors as visualized by significantly reduced staining for Ki67 (Fig. 3d and Supplementary Figure 7b). In skin tumors, the direct proportionality between tumor proliferation and tumor size is complicated by the different cell types within the tumor mass. In fact, LGK974-treated tumors display enhanced differentiation/keratinization, which complicates the assessment of the actual tumor size. Furthermore, LGK974-treated tumors are more differentiated and thus contain an increased amount of keratinized matter (Fig. 3c) formed by extracellular matrix and keratin, which is dead material.

Together, our findings indicate that blocking WNT secretion promotes differentiation, and reduces proliferation as well as invasive potential of established HPV-driven cSCC.

### PORCN inhibition decreases the malignant potential of HPV-driven cSCCs

To clarify the downstream effects of PORCN inhibition in HPV-driven cSCCs, we performed RNA sequencing of LGK974- and vehicle-treated tumors (Supplementary Figure 7c). Gene ontology analysis [25] of the top deregulated transcripts (*p* < 0.05) showed “regulation of keratinocyte migration” as the primary hit, which confirms our findings of reduced invasiveness and malignancy of the tumors upon LGK974 treatment (Supplementary Figure 7d). In particular, we observed a significant downregulation of transcripts for matrix metalloproteinases (*Mmp9*, *Mmp10*, *Mmp13*) and upregulation of the metalloproteinase inhibitor *Timp4* [43] (Supplementary Figure 7e). As expected from Wnt pathway inhibition, we did not find indications of a reduction in MAPK signaling. Because we performed RNA sequencing on whole tumor tissues, we cannot draw conclusions about whether LGK974 treatment affects tumor cells, tumor-infiltrating cells or both. To confirm some findings from the RNA sequencing and to clarify in which cell types the changes occur, we performed immunohistochemistry for HMGA2 and MMP13. Our observation that LGK974 treatment reduced the expression of HMGA2 in tumor cells suggests a tumor-intrinsic effect of PORCN inhibition (Fig. 3e). The stark reduction of MMP13 in tumor as well as stromal tissue upon LGK974 administration (Fig. 3e) suggests that PORCN inhibition also affects stromal cells.

To investigate whether PORCN inhibition influences CSCs, we analyzed the cellular composition of control and LGK974-treated tumors by flow cytometry. Blocking WNT secretion resulted in the reduction of the CD34^+^ EpCAM^+^ CD31^−^ CSC population [19], (Fig. 3f and Supplementary Figure 7f). This was confirmed by antibody staining for CD34 on tumor sections (Supplementary Figure 7g), again underscoring the importance of WNT secretion for CSC maintenance and thus tumor growth and malignancy.

We investigated the pathways involved in tumorigenesis and growth of cSCCs that are driven by HPV. Our finding that WNT signaling is crucial for tumorigenesis is in line with work on chemically induced cSCCs [14]. Of note, in the case of cSCCs no prominent activating mutations downstream of the WNT/β-catenin pathway components were found [6], in contrast to classical WNT/β-catenin-dependent cancers like colon carcinomas [10]. WNT/β-catenin activation in HPV-driven cSCC seems rather to be reached by upregulation of WNTs and factors such as PORCN that are required for their secretion. The combination is apparently crucial to maintain a proper CSC niche. Compelling evidence in support of this theory comes from a study by [30], where they show that the CSC niche of lung adenocarcinomas is maintained by cells expressing high levels of PORCN. By using PORCN inhibitor on HPV-driven cSCCs, we show the addiction of these cancers for WNT secretion. As inhibiting WNT secretion severely hampers tumorigenesis, PORCN inhibition might thus be a valuable preventive measure against cSCCs after organ transplantation and subsequent immunosuppression. The fact that not only cSCCs, but also lung adenocarcinomas seem to upregulate PORCN to maintain a CSC niche asks for further investigation into this matter in further epithelial tumor types. It also draws attention towards the necessity to not only check the mutational landscape in tumors but also to carefully check changes in the transcriptome.

In conclusion, our results provide novel insights into the molecular details of HPV-driven cSCCs. They also reveal a possible therapeutic approach to prevent and treat this type of cancers.

## Electronic supplementary material

Supplementary Figure 1

Supplementary Figure 2

Supplementary Figure 3

Supplementary Figure 4

Supplementary Figure 5

Supplementary Figure 6

Supplementary Figure 7

Supplementary Figure Legends
