# Supplementary figures and images for: WNT ligands control initiation and progression of human papillomavirus-driven squamous cell carcinoma
Source: Oncogene. 2018 Apr 17;37(27):3753–62. doi: 10.1038/s41388-018-0244-x (PMC6033839; doi:10.1038/s41388-018-0244-x)

Supplementary Figure 1

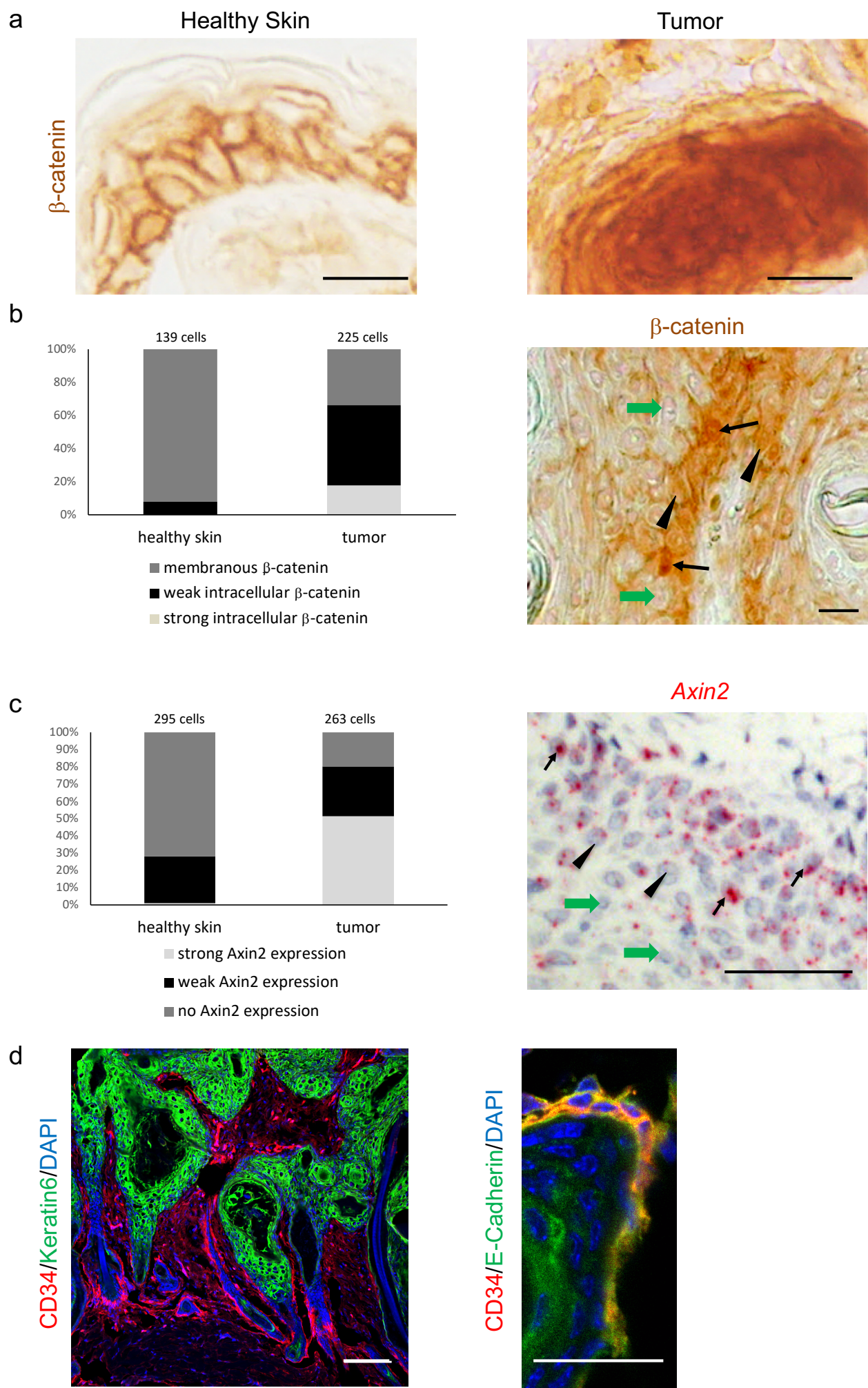

Supplement: Supplementary file 1 — Supplementary Figure 1 [file 41388_2018_244_MOESM1_ESM.pdf]

Supplementary Figure 3

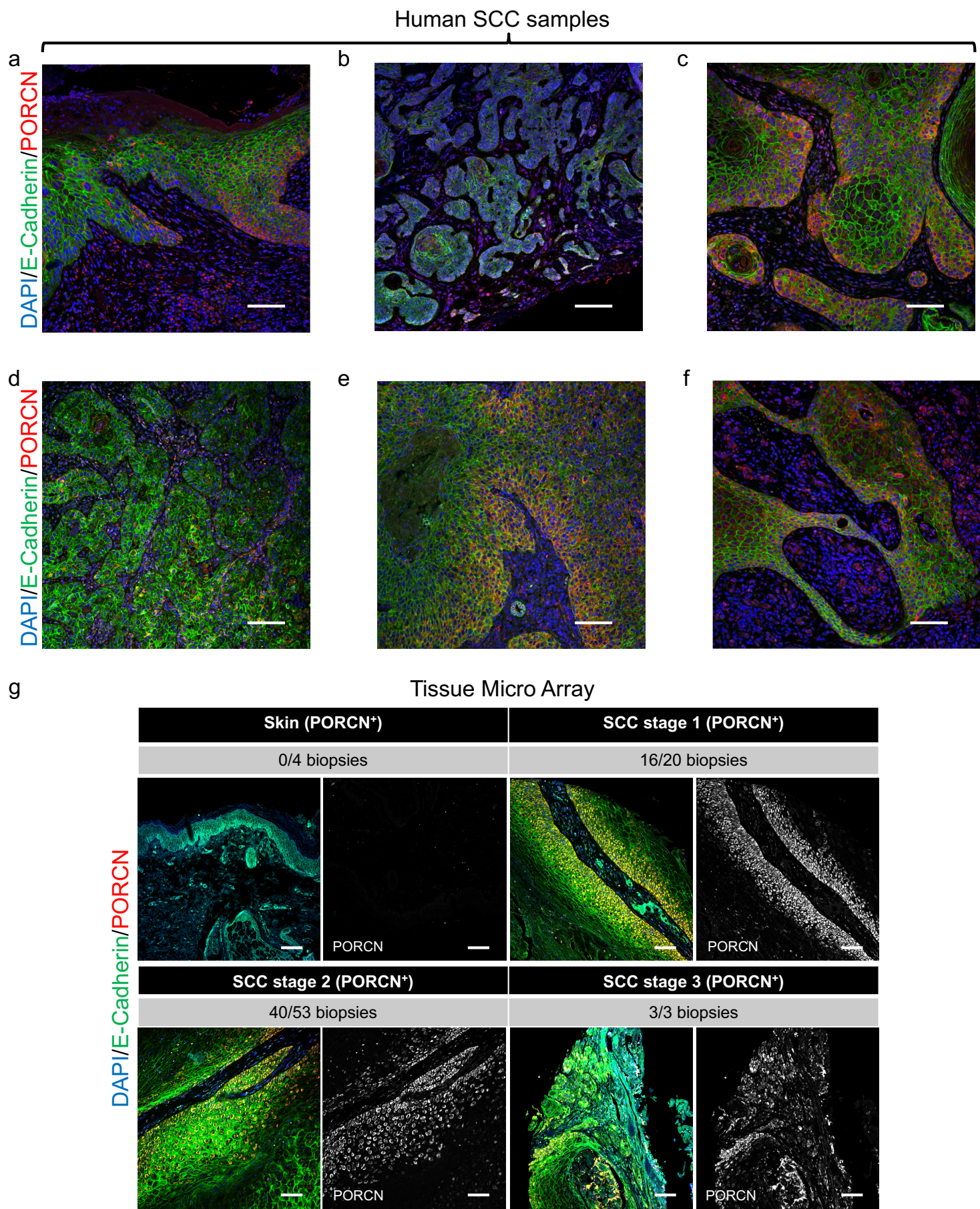

Supplement: Supplementary file 3 — Supplementary Figure 3 [file 41388_2018_244_MOESM3_ESM.pdf]

Supplementary Figure 4

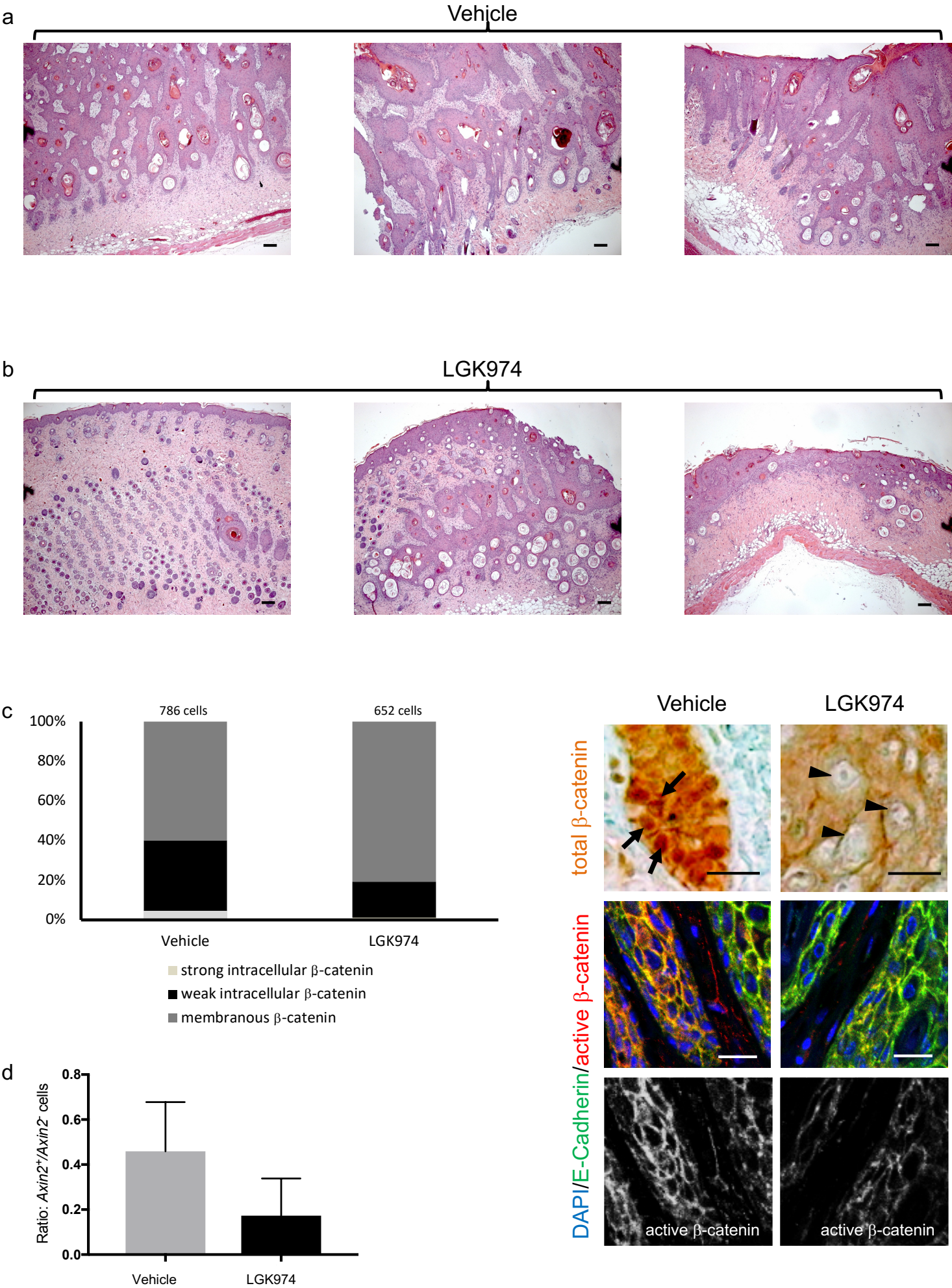

Supplement: Supplementary file 4 — Supplementary Figure 4 [file 41388_2018_244_MOESM4_ESM.pdf]

Supplementary Figure 5

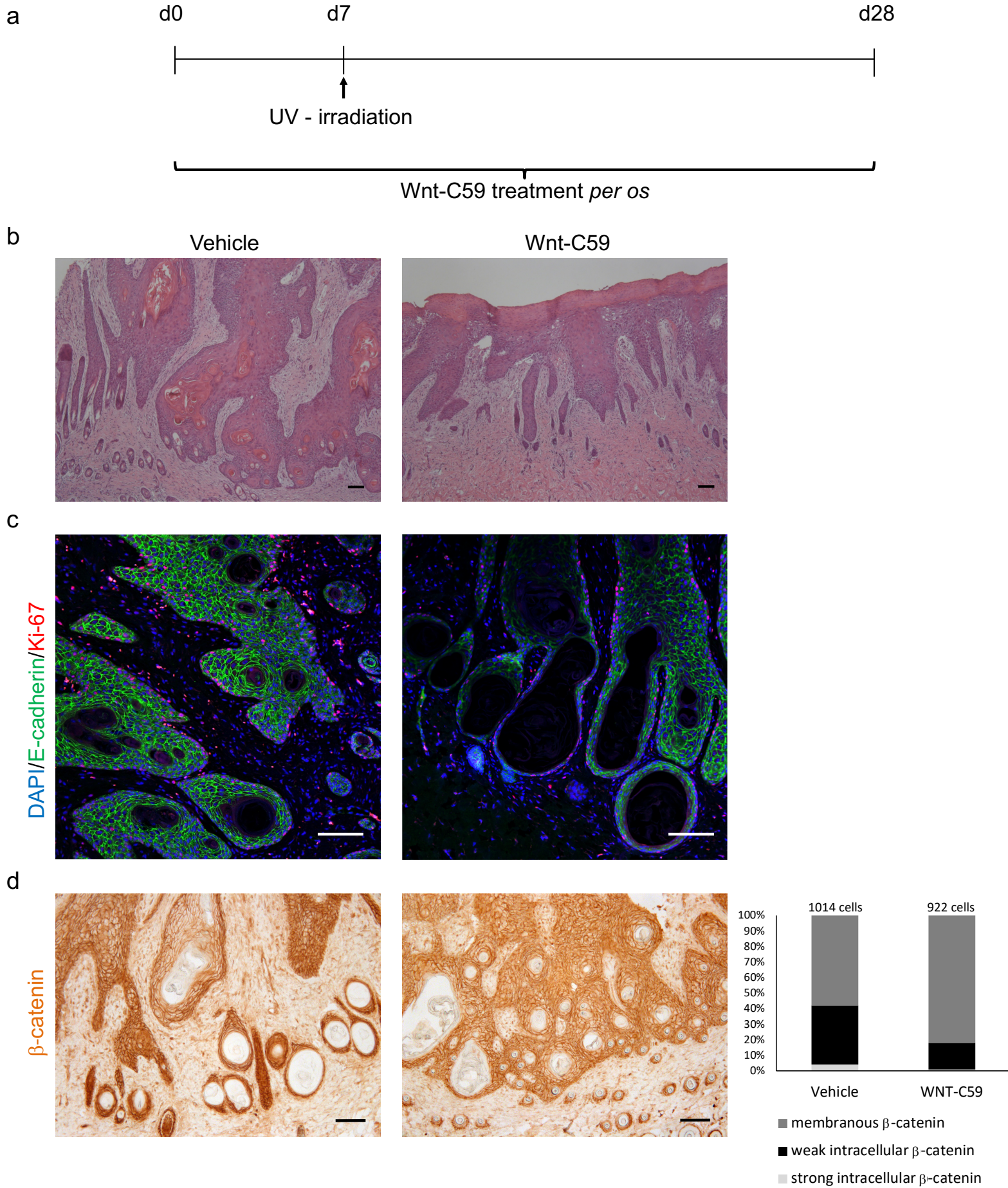

Supplement: Supplementary file 5 — Supplementary Figure 5 [file 41388_2018_244_MOESM5_ESM.pdf]

Supplementary Figure 6

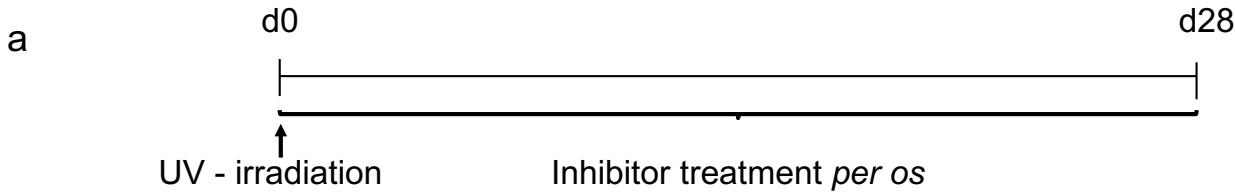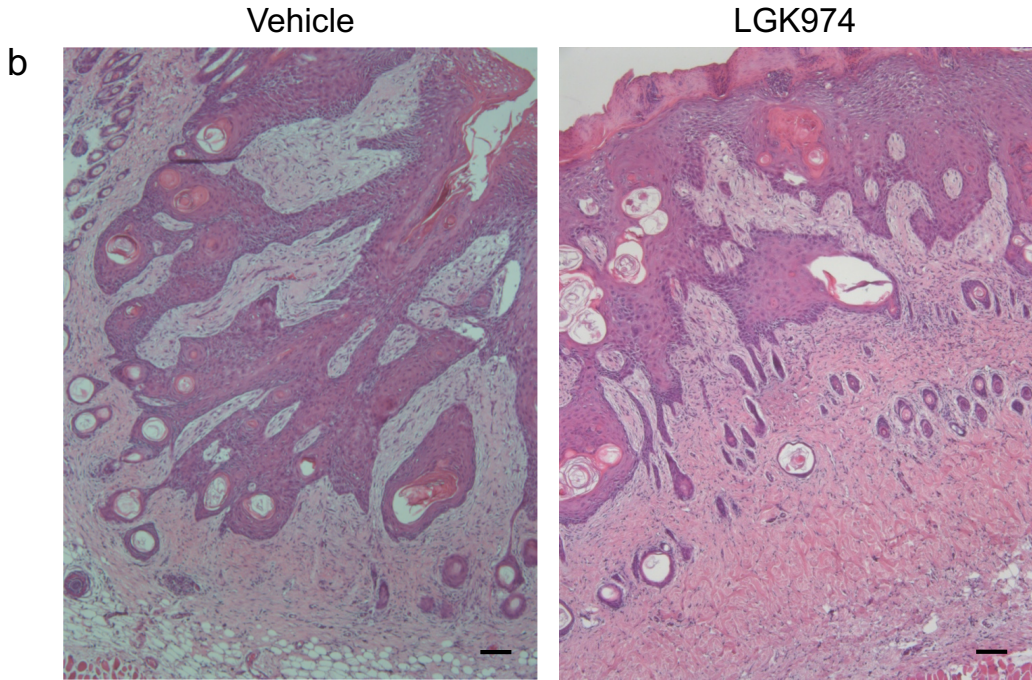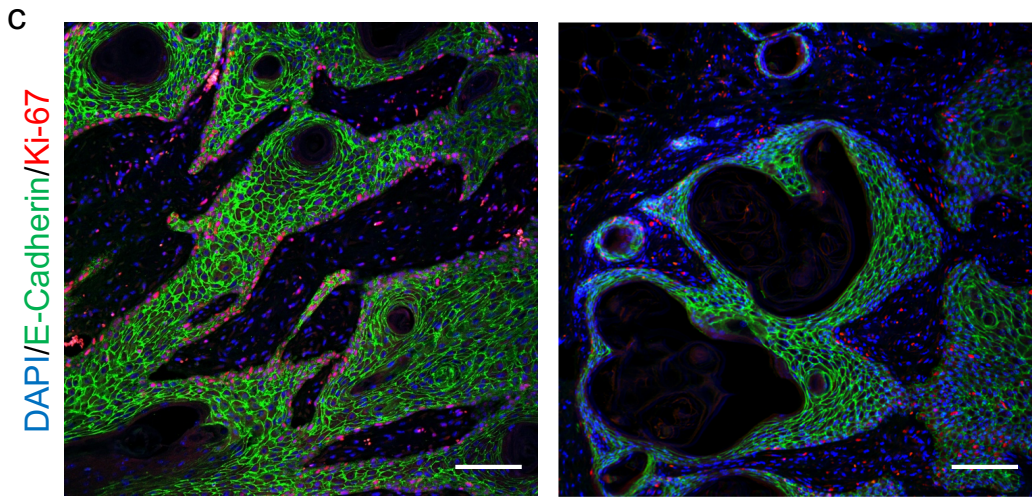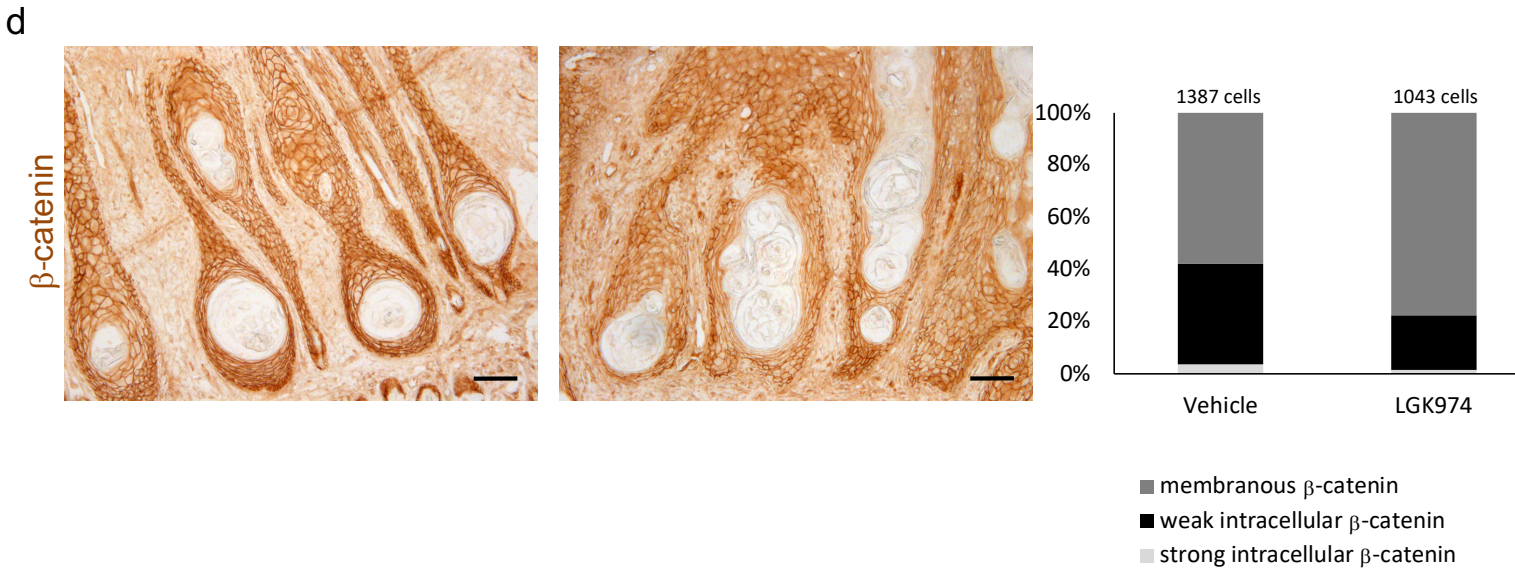

Supplement: Supplementary file 6 — Supplementary Figure 6 [file 41388_2018_244_MOESM6_ESM.pdf]

## Supplementary Figure 7

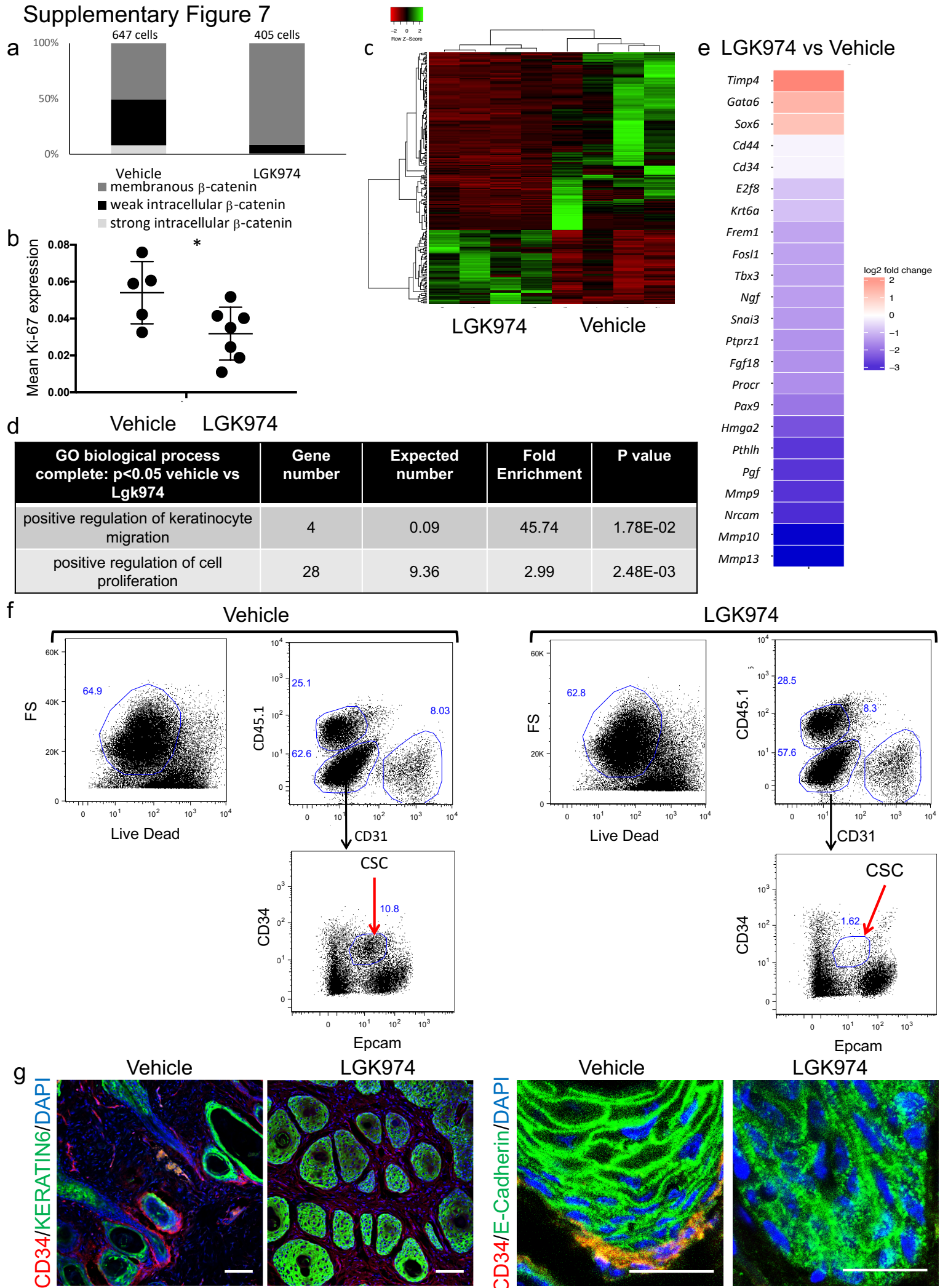

Supplement: Supplementary file 7 — Supplementary Figure 7 [file 41388_2018_244_MOESM7_ESM.pdf]
