# Supplementary material for: WNT ligands control initiation and progression of human papillomavirus-driven squamous cell carcinoma
Source: Oncogene. 2018 Apr 17;37(27):3753–62. doi: 10.1038/s41388-018-0244-x (PMC6033839; doi:10.1038/s41388-018-0244-x)

Supplementary Figure 2

a

Selected GO terms of first 1500 hits skin vs tumor

GO biological process complete	Gene number	Expected number	Fold Enrichment	P value
positive regulation of MAPK cascade	66	29.91	2.21	4.54E-05
regulation of cell migration	98	47.94	2.04	5.45E-07
inflammatory response	63	27.09	2.33	1.74E-05

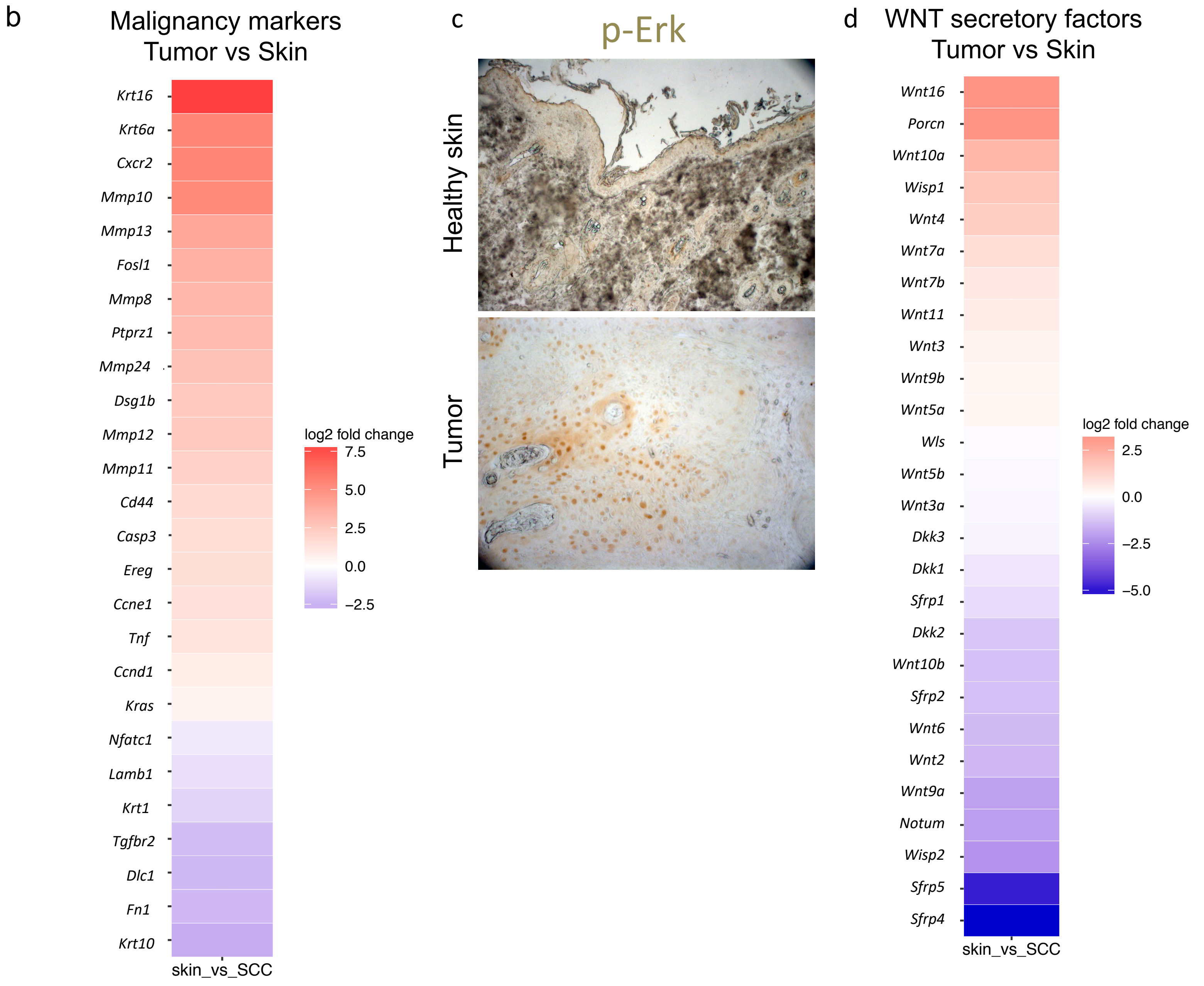

Supplement: Supplementary file 2 — Supplementary Figure 2 [file 41388_2018_244_MOESM2_ESM.pdf]
