# Supplementary material for: WNT ligands control initiation and progression of human papillomavirus-driven squamous cell carcinoma
Source: Oncogene. 2018 Apr 17;37(27):3753–62. doi: 10.1038/s41388-018-0244-x (PMC6033839; doi:10.1038/s41388-018-0244-x)
Supplement: Supplementary file 8 — Supplementary Figure Legends [file 41388_2018_244_MOESM8_ESM.docx]

**SUPPLEMENTARY FIGURE LEGENDS**

**Supplementary Figure 1: Secretion of WNTs is elevated in HPV-driven cSCCs in comparison to healthy skin, leading to active WNT/β-catenin signaling. Relates to Figure 1.**

(a) Intracellular (cytoplasmic and nuclear) accumulation of β-catenin in HPV-driven SCC tumor epithelial cells (right panel) was in contrast to membrane localization of β-catenin in keratinocytes of healthy skin (left panel). High magnification micrographs corresponding to Figure 1a. Scale bar = 50 μm.

(b) Quantification of membranous and intracellular β-catenin in HPV-driven SCC tumor epithelial cells and keratinocytes of healthy skin revealed increased number of cells with intracellular β-catenin. Left panel: Individual cells in equal areas from representative images of tumor tissue (255 cells) and healthy skin (139 cells) were evaluated with respect to the expression of β-catenin. The proportion of cells corresponding to one of the three phenotypes (strong intracellular β-catenin, weak intracellular β-catenin, membranous β-catenin) is displayed. Right panel: Representative image used for the quantification of the β-catenin distribution. Black arrows point to cells with strong intracellular β-catenin expression, black arrowheads show cells with weak intracellular β-catenin stabilization, green arrows point to cells with solely membranous β-catenin expression. Scale bar = 20μm.

(c) Quantification of *Axin2* mRNA expression determined by ISH. Left panel: Individual cells in equal areas from representative images of tumor tissue (263 cells) and healthy skin (295 cells) were evaluated with respect to the expression of *Axin2*. The proportion of cells corresponding to one of the three phenotypes (no, weak or strong *Axin2*) is displayed. Right panel: Black arrows point to cells with strong *Axin2* expression, black arrowheads show cells with weak *Axin2* expression, green arrows point to cells where *Axin2* was not detected. Scale bar = 100μm.

(d) Left panel: Representative section showing expression of CD34 (red) in HPV-driven cSCC at the tumor-stroma interphase in the invasive front of the tumors. Nuclei are counterstained with DAPI (blue), Keratin 6 (green) marks epithelial tumor cells. Scale bar = 100 μm. Right panel: CD34 (red) and E-Cadherin (green) co-staining in HPV-driven SCC indicates the tumor epithelial origin of CD34 positive cells. The orange color indicates co-expression of E-Cadherin and CD34, marking those cells as putative CSCs. Tumors were isolated 4 weeks after induction. Scale bar = 25μm.

**Supplementary Figure 2: Enhanced Secretion of WNTs is connected with altered gene expression in HPV-driven cSCCs in comparison to healthy skin. Relates to Figure 1.**

(a) Gene ontology analysis of RNA-sequencing data comparing HPV-driven cSCCs with healthy skin. Four biological replicates for each condition were sequenced and analyzed.

(b) Heatmap showing expression of selected malignancy-associated genes in cSCC compared to healthy skin. Malignancy markers are upregulated, while differentiation markers are downregulated in cSCC.

(c) Staining for phosphorylated ERK (p-ERK, brown) in HPV-driven cSCC four weeks after tumor induction. P-ERK was not detected in healthy skin.

(d) Heatmap showing expression of transcripts for *Wnts* and factors implicated in WNT secretion or availability in cSCC compared to healthy skin; tumor tissue shows increased expression of transcripts associated with WNT production and decreased expression of transcripts for WNT inhibitory factors.

For RNA-sequencing, RNA was isolated from healthy skin (n=4 mice) and tumors from LGK974-treated (n=4 mice) and vehicle-treated (n=4 mice) mice using TRI-Reagent combined with the Ambion PureLink RNA mini Kit. Each sample was obtained from an individual mouse. Single-stranded 100-bp read sequencing was performed on an Illumina HiSeq2500 machine at the Genomics Platform of the University of Geneva. The reads were mapped with the TopHat v.2 software to the Ensembl GRCm38.p4 reference on new junctions and known junction annotations. Biological quality control and summarization were performed with PicardTools1.141. EdgeR was used for normalization and differential expression testing. Raw data are deposited here: GEO database, accession code is GSE108454.

Clustering analysis was performed using the online tool described in (45), other heatmaps were generated in R-studio. The top-ranked genes for the clustering analysis and other heatmaps were chosen according to p<0.05 (general linear model with negative binomial distribution, (quasi-likelyhood F-test)). False discovery rate was too stringent due to the high variability between different tumors and low number of changing transcripts. qRT-PCR and immunohistochemistry was used to confirm the findings.

Protocols not mentioned here are described in the legends of Figure 1.

**Supplementary Figure 3: Human cSCCs show PORCN expression at the invasive front. Relates to Figure 1.**

(a-f) Staining of six human cSCC for PORCN (red), E-cadherin (green) and DAPI (blue). Each picture shows an individual patient with dedifferentiated (a, b), moderately differentiated (c, d, e) or well-differentiated (f) cSCC as assessed by a pathologist.

(g) A commercially obtained tissue microarray of human SCCs (US Biomax, array SK802b) was stained for PORCN (red), E-cadherin (green) and counterstained with DAPI. The TMA contained 4 samples from healthy human skin, 20 samples from stage 1 SCC, 53 samples from stage 2 SCC and 3 samples from stage 3 SCC. PORCN-positive epithelial cells are visible as yellow cells (left panels) and as white cells (in right single-color panels). The proportion of biopsies with PORCN-positive cells is given in the grey boxes above representative examples. Scale bar = 100 μm. Protocols are described in the legend of the main figures.

**Supplementary Figure 4: Tumors are replaced by hair follicles and keratin in mice treated with pre-emptive LGK974. Relates to Figure 2.**

(a) Three representative H&E stained sections from individual mice treated with vehicle prior to UV-irradiation to induce tumors (see Figure 2a). The group contained 5 mice.

(b) Three representative H&E stained sections from individual mice treated with LGK974 prior to UV-irradiation to induce tumors (see Figure 2a). The group contained 6 mice.

(c) Quantification of β-catenin expression in LGK974- and vehicle-treated HPV-driven SCCs reveals a decrease in intracellular β-catenin upon inhibition of PORCN. Left panel: Quantification as described in the legends to Supplementary Figure 1b. Right panel: Upper panels show high magnification images of β-catenin staining of vehicle-treated (left) and LGK974-treated (right) tumors. Arrows point to cells with nuclear/cytoplasmic β-catenin, arrowheads to cells without any intracellular β-catenin. Scale bar =20 μm.

Middle and lower panels show a co-staining of E-cadherin (green) and active (unphosphorylated; antibody clone D13A1 obtained from Cell Signaling Technology) β-catenin (red) of vehicle-treated (left) and LGK974-treated (right) tumors. Representative images indicate reduced active β-catenin upon PORCN inhibition. Scale bar = 25 μm.

(d) PORCN-inhibition results in a decreased proportion of *Axin2-*expressing SCC cells. Quantification was performed as described in the legends to Figure 3b.

Protocols are described in the legend of Figures 1 and 2.

**Supplementary Figure 5: Pre-emptive PORCN inhibition by the inhibitor Wnt-C59 reduces tumor size, thus confirming the specificity of the results obtained by LGK974 usage. Relates to Figure 2.**

(a) Experimental design. Treatment with Wnt-C59 (application by oral gavage, 10 mg/kg diluted in citrate buffer, pH 3) or vehicle was started 7 days prior to tumor induction by UV-irradiation. Mice were treated daily until the endpoint at day 28. Experimental groups consisted of 4 mice. The experiment was performed once.

(b) Representative H&E-stained sections of vehicle-treated (left panel) and Wnt-C59-treated (right panel) treated tumors.

(c) Representative staining for Ki-67 (red) indicates reduced proliferation in Wnt-C59-treated SCC. E-cadherin (green) was used to delineate cell outlines, DAPI (blue) to mark the nuclei.

(d) Representative β-catenin staining confirms the ability of the alternative PORCN-inhibitor Wnt-C59 to reduce intracellular accumulation of β-catenin and thus Wnt/β-catenin signaling. Left panels: Representative staining. Right panel: Quantification of intracellular and membranous staining of β-catenin as described in the legends to Supplementary Figure 1b.

Scale bars = 100 μm. Protocols are described in the legends of Figures 1 and 2.

**Supplementary Figure 6: Inhibition of PORCN by LGK974 from the time of tumor induction onwards reduces tumor growth. Relates to Figure 2.**

(a) Experimental design. Treatment with LGK974 (6 mg/kg per oral gavage) was started at the time of tumor induction by UV-irradiation. Mice were treated daily until the endpoint at day 28. Control mice were treated with vehicle. The vehicle-treated group consisted of 4, the LGK974-treated group of 5 mice. The experiment was performed once.

(b) Representative H&E staining of vehicle-treated (right panel) and LGK974-treated (left panel) tumors.

(c) Representative staining for Ki-67 (red) indicates reduced proliferation in LGK974-treated SCC. E-cadherin (green) was used to delineate cell outlines, DAPI (blue) to mark the nuclei.

(d) Representative β-catenin staining shows the ability of the PORCN-inhibitor LGK974 to reduce intracellular accumulation of β-catenin and thus Wnt/β-catenin signaling. Left panels: Representative staining. Right panel: Quantification of intracellular and membranous staining of β-catenin as described in the legends to Supplementary Figure 1b. Scale bar = 100 μm. Protocols are described in the legends of Figures 1 and 2.

**Supplementary Figure 7: Treatment of HPV-driven cSCC with LGK974 reduces proliferative and invasive capacity of the tumors. Relates to Figure 3.**

(a) Treatment with LGK-974 reduced intracellular accumulation of β-catenin in HPV-driven SCC. Quantification of intracellular and membranous β-catenin of tumors from the experiment shown in Figure 3b as described in the legends to Supplementary Figure 1b.

(b) Proliferation in epithelial areas of 6 LGK974- and 5 vehicle-treated tumors indicated by Ki-67 staining (Figure 3d) and quantified using the Vectra 3.0 system (PerkinElmer). Symbols represent individual mice.

(c) Clustering analysis (average linkage, Pearson distance measurement) showing the differentially expressed genes (general linear model with negative binomial distribution, (quasi-likelyhood F-test) **p*<0.05) from an RNA-sequencing experiment comparing four LGK974- with four vehicle-treated tumors.

(d) Gene ontology analysis of RNA-sequencing data comparing vehicle- and LGK974-treated HPV-driven cSCCs. Four independent samples for each condition were sequenced and analyzed. Raw data are deposited here: GEO database, accession code is GSE108454.

(e) Heatmap showing selected differentially expressed genes associated with SCC malignancy by RNA-sequencing of 4 LGK974- and 4 vehicle-treated tumors.

(f) Flow-cytometric analysis of 6 LGK974- and 5 vehicle-treated tumors. Cells were stained for live/dead, CD45.1 (leukocytes), EpCAM (epithelial cells including tumor), CD31 (vascular endothelial cells) and CD34 (CSC). The EpCAM^+^ CD34^+^ CSC population is clearly reduced upon treatment (see also Figure 3f).

(g) Left panels: Staining for CD34 (red) and Keratin-6 (green) in LGK974- and vehicle-treated tumors. CD34 staining is lost upon treatment along the tumor-stroma interphase. Sections are counterstained with DAPI (blue). Scale bar = 100 μm. Right panels: CD34 (red) and E-Cadherin (green) co-staining in LGK974 and vehicle treated tumors in high magnification, confirming the loss of co-expression of these markers upon LGK974 treatment. Scale bar = 20μm. Tumors were isolated 4 weeks after induction.

This experiment was performed twice with similar results. The protocols are described in the legend of Figures 1 and 2.
